# Indigenous Autism in Canada: A Scoping Review

**DOI:** 10.1007/s10803-023-06045-z

**Published:** 2023-07-22

**Authors:** Grant Bruno, Titus A Chan, Lonnie Zwaigenbaum, Emily Coombs, The Indigenous Relations Circle, David Nicholas

**Affiliations:** 1 Department of Pediatrics, Edmonton Clinic Health Academy, University of Alberta, 11405- 87 Avenue, Edmonton, AB T6G 1C9 Canada; 2 Samson Cree Nation, Maskwacîs, AB Canada; 3 Toronto, ON Canada; 4 Department of Pediatrics, University of Alberta c/o Glenrose Rehabilitation Hospital, 10230 111th Avenue, Edmonton, AB T5G 0B Canada; 5 Department of Educational Psychology, Counselling Psychology, University of Calgary, 2500 University Drive NW, Calgary, AB T2N 1N4 Canada; 6 The Autism Society of Alberta, Calgary, Canada; 7 Faculty of Social Work, University of Calgary, 2500 University Drive NW, Calgary, AB T2N 1N4 Canada

**Keywords:** Indigenous, Autism, Canada, Scoping Review, Indigenous Quality Assessment Tool (IQAT)

## Abstract

Currently there is a severe lack of research on autism and Indigenous people in Canada. This scoping review explores this literature gap and assesses the same literature from an Indigenous perspective. Scoping reviews are an effective means to explore the literature in a specific area, in this case, autism and Indigenous people in Canada. We explored existing literature as it pertains to Indigenous populations and autism in Canada. To support this review, the Indigenous Quality Assessment Tool (QAT) was adapted to appraise the quality of literature. In total, there were a total of 212 articles identified of which 24 met the inclusion criteria: (1) some focus on autism, (2) a component specific to Indigenous people, and (3) specific to Canada. Of the 24 articles and reports, 15 were peer-reviewed and the rest considered grey literature. Most articles focused on program delivery with some literature using primary data (quantitative and/or qualitative). Overall, the quality of the research was appraised as poor, as determined by the QAT. Findings reaffirm the critical need for research that addresses autism in Indigenous communities within Canada and show the importance of having research done in full partnership with, or led by, Indigenous people.

## Introduction

### Rationale

The DSM-5 characterizes autism as being associated with difficulties with communication, social deficits, and restricted, repetitive behaviors and interests (American Psychiatric Association, 2013). It is widely recognized that autism occurs in all populations, including Indigenous populations. The prevalence of autism in Indigenous communities in Canada is currently unknown and there is minimal academic literature on lived experience related to autism in cultural contexts within Canada (Lindblom, 2014). Autism research in Canada disproportionately focusses on the biomedical and clinical aspects of autism rather than the public health or social aspects (Krahn & Fenton, 2012).

Canada’s Constitution recognizes three distinct Indigenous groups, First Nations, Métis, and Inuit. There are 1.8 million Indigenous people, accounting for 5.0% of the total population in Canada (Statistics Canada, 2021), including 600 First Nations with 70 different languages as well as numerous Métis groups and 50 Inuit groups. The experiences of Indigenous peoples are complex and unique. Their experiences in Canada have been defined and influenced by *Sect. 35 of Canada’s Constitution*, geography, legal status, and other colonial experiences in multiples contexts such as the child welfare, education, and justice systems.

Indigenous peoples in Canada have been subjected to racism and systemic inequities which have resulted in poorer health outcomes, social inequalities, and poorer access to services – lingering and continuing impacts of destructive colonial legislation and policies. Indigenous populations in Canada have significantly higher rates child mortality, maternal morbidity, infectious disease, shorter life expectancy, higher rates of malnutrition, substance abuse, lifestyle-related chronic diseases and conditions, accidents, homicide, violence, and suicides than the mainstream population (Barnabe, 2021; Gracey & King, 2009; Smylie & Anderson, 2006; Wilson & Young, 2008). Colonial policies have had severe and long-standing impacts. For instance, the *Indian Act of 1876* outlawed cultural practices, including ceremonies, and enacted the residential school system. Such policy and widespread practices are still being felt in First Nations communities across Canada today (Wilk et al., 2017). Furthermore, the *Indian Act* stipulates that the federal health minister has overarching power over ‘mentally incompetent Indians’ (Bartlett, 1977), which could be extrapolated even today to include Autistic Indigenous people. Such language and perspectives and the principles beneath this terminology raise concern about presumptions, imposed power and potential impacts. Finally, First Nation reservations are under the *Indian Act* and are considered federal jurisdiction whereas disability services, including autism services, are a provincial responsibility, both levels of government often pass the obligation on to each other leaving reservations without any services.

To our understanding this scoping review is the first to explore the literature on Indigenous people and autism in the context of Canada from an Indigenous perspective. The research questions below were developed with the intention to allow for a broad search of the literature while also being detailed enough to be able to search within a reasonable scope.

#### Research Questions

1. What is known about autism in Indigenous communities in Canada based on the literature?
2. What are the areas of focus in published research on autism and Indigenous peoples in Canada?
3. What is the quality of research on autism in Indigenous communities in Canada?

### Terminology

Terminology in both Indigenous and autistic communities are evolving and dynamic. *Indigenous* is a catch all term that is appropriate in some cases such as when describing broad concepts or ideas; however, when working with communities it is best to be as specific as possible, such as indicating tribal or community membership. The terms Aboriginal, Native, Indian are outdated and only used in some instances, such as when exploring historical literature or legal definitions such as ‘Status Indian’.

Autism also has an evolving terminology (Monk et al., 2022). In this review, we use identity first language such as ‘Autistic person’ rather than ‘person with autism’. We will also be using the term ‘autism’ rather than ‘autism spectrum disorder’ sometimes referred to as ASD.

## Methods

Scoping reviews are used to identify and aggregate available evidence in each field. These reviews clarify key concepts and definitions within the field of inquiry, examine how research is conducted, and identify key characteristics or factors related to a concept, and finally analyze knowledge gaps (Munn et al., 2018).

Arksey and O’Malley (2005) identified the following five stages in a scoping review: (1) determining the research question, (2) identifying relevant studies, (3) study selection, (4) charting the data, and (5) collating, summarizing and reporting the results. Some reviews assess quality and we have adopted this sixth stage; methodologic/research rigor was analyzed using the Indigenous Quality Assessment Tool (IQAT) (Harfield et al., 2020). For this review, we will be using the PRISMA Extension for Scoping Reviews (PRISMA-ScR) through Covidence (Tricco et al., 2018).

### Protocol and Registration

A scoping review protocol was developed and registered with Open Science Framework. The protocol is titled, “Indigenous Autism in Canada: A Scoping Review” (Bruno, 2022).

### Inclusion and Exclusion Criteria

The following inclusion and exclusion criteria were adopted. Inclusion/exclusion decisions were determined independently by the two reviewers and if there was disagreement, the two reviewers met to resolve this via consensus.

#### Inclusion

We included articles or reports that have a component specific to *Indigenous peoples* (First Nations, Métis, Inuit) and *disability*, with some focus on autism. Articles and reports had to be located within Canada or have a significant component about the Canadian context. Articles and reports relating to Indigenous people in Canada and disability more generally, that mentioned autism within the report, were also included, as were academic theses’ on this research topic.

#### Exclusion

Articles and reports that did not include a specific focus on autism (e.g., studies that exclusively focused on other developmental disabilities in Indigenous populations) were not included. Articles and reports that addressed autism but without substantial focus on Indigenous populations were also excluded. Articles and reports in which non-Indigenous and Indigenous autistic participants could not be differentiated were removed. Publications that mentioned Indigenous populations in Canada and autism but did not detail the context were excluded. This included missing demographic factors such as what Indigenous population was studied (e.g., First Nations, Métis, or Inuit). Conference abstracts, book reviews, news/media reports and opinion pieces were excluded.

### Information Sources & Search

A health sciences librarian assisted in developing a search strategy and identifying keywords and databases relevant to our research questions. As suggested by Arksey and O’Malley (2005), we were flexible and iterative in using search terms to create our final search strategy. The aim was to capture the broadest possible selection of papers in this area of study. The search terms represented associated keyword combinations of autism, Indigenous communities, and Canada (see Table 1).

**Table 1:** Search terms used

Concept	Keywords used
Autism	Autis*, Asperge*, ASD, PDD, PDD-NOS, pervasive development disorder, childhood disintegrative disorder, autistic disorder, Kanner
Indigenous	Indigenous, Aboriginal, Premières Nations, First Nations, Metis, on-reserve, off-reserve, Athapaskan, Saulteaux, Wakashan, Cree, Dene, Inuit, Inuk, Inuvialuit, Haida, Ktunaxa, Tsimshian, Gitsxan, Nisga’a, Haisla, Heiltsuk, Oweenkeno, Kwakwaka’wakw, Nuu chah nulth, Tsilhqot’in, Dakelh, Wet’suwet’en, Sekani, Dunne-za, Dene, Tahltan, Kaska, Tagish, Tutchone, Nuxalk, Salish, Stl’atlimc, Nlaka’pamux, Okanagan, Secwépemc, Tlingit, Anishinaabe, Blackfoot, Nakoda, Tasttine, Tsuu T’inia, Gwich’in, Hän, Tagish, Tutchone, Algonquin, Nipissing, Ojibwa, Potawatomi, Innu, Maliseet, Mi’kmaq, Micmac, Passamaquoddy, Haudenosaunee, Cayuga, Mohawk, Oneida, Onodaga, Seneca, Tuscarora, Wyandot
Canada	Canad*, North Americ*, British Columbia, Alberta, Saskatchewan, Manitoba, Ontario, Quebec, Nova Scotia, New Brunswick, Newfoundland, Labrador, Prince Edward Island, Yukon Territory, NWT, Northwest Territories, Nunavut, Nunavik, Nunatsiavut, NunatuKavu

*Denotes truncation/stemming

We performed an extensive systematic search to find articles within the specified criteria. Databases searched comprise: MEDLINE, CINAHL, ERIC, PsycINFO, Web of Science, Cochrane Library, PubMed, and Google Scholar. Additionally, the following North American Indigenous research databases were searched: iPortal (Indigenous Studies Portal - University of Saskatchewan), Circumpolar Health Bibliographic Database, Bibliography of Native North Americans, and Native Health Database. Each database was queried using Boolean or database-specific operators. Database searches were limited to articles published in English between 2000 and 2022.

### The Selection of Sources of Evidence

The study selection process was based on the strategy outlined by Arksey and O’Malley (2005) and the Preferred Reporting Items for Systematic Reviews and Meta-Analyses extension for Scoping Reviews (PRISMA-ScR) framework (Tricco et al., 2018). All references and abstracts were imported and screened using *Covidence*, a web-based software for systematic and scoping reviews that supports and organizes review content. Authors, GB and TC, independently reviewed all sources in two stages as follows: title and abstract review for inclusion, and if included, full text review. Covidence automatically generated Cohen’s kappa coefficients (κ) to determine interrater agreement. Proportional agreement, the extent to which reviewers similarly rated article inclusion/exclusion, was recorded in Covidence. Any discrepancies between reviewers on the eligibility/ineligibility of an article were discussed and resolved through consensus.

### Data Charting Process

With initial guidance of a librarian for the search strategy, data was compiled and charted for synthesis by GB and TC through Covidence. In this data charting process, the reviewers remained in close contact to compare emergent rankings and observations in the selected documents and met regularly to discuss findings. A data extraction template was created to extract the following information from each article and report that was reviewed: author(s), year of publication, type of publication, was the article peer-reviewed, Indigenous authorship, Indigenous involvement, study focus, study design, study funding. Data on socioeconomic status and educational attainment levels were not recorded.

### Indigenous Quality Assessment Tool

The Indigenous Quality Assessment Tool (IQAT) is a standardized instrument that examines research pertaining to Indigenous peoples (Harfield et al., 2020). Fourteen questions are used to appraise study quality from an Indigenous perspective particularly with respect to research governance, community engagement, regard for cultural and intellectual property, and capacity building (see Table 3). Originally developed in an Australian context the IQAT was adapted from ‘Aboriginal and Torres Strait’ to ‘Indigenous’ to fit the context within Canada. It was integrated as a part of the review process to assess the extent to which and how Indigenous people had been engaged and included in each article. While the IQAT is a relatively new research instrument, it provides valuable insight into the quality and process of research that has taken place. Two reviewers GB and TC independently appraised each article using the adapted IQAT and resolved any disagreements through consensus. Studies were assessed as “high quality” if they met at least 10 of the 14 appraisal questions, “moderate quality” if meeting 6 to 9 out of 14 questions, and “low quality” if meeting 5 or less questions, based on previous applications of the IQAT in literature (Christidis et al., 2021).

### Community Involvement

The lead author (GB) is Nehiyaw (Plains Cree), a registered member of Samson Cree Nation, one of the reserves that makes up Maskwacîs, a parent to Autistic children, and a PhD Medical Sciences candidate in the Department of Pediatrics at the University of Alberta. TC is a non-Indigenous settler, with experience as a researcher and registered social worker with family caregivers. LZ is a non-Indigenous Professor in Pediatrics at the University of Alberta and director of the Autism Research Center at the Glenrose Rehabilitation Hospital. DN is non-Indigenous and a Professor in Social Work at the University of Calgary. EC is a racially ambiguous Autistic graduate student. This review was also brought to the Autism Society of Alberta’s Indigenous Relations Circle for their feedback and guidance. This Circle is made up of Indigenous Autistics, parents and caregivers to Autistic children, autism service providers, autism researchers, and those with an interest, professional, personal or both, in autism. The Circle meets monthly to discuss autism in Indigenous communities and uses those learnings to advocate as well as plan cultural events for Indigenous Autistics and their families. In this case, the Circle guided and endorsed this review process.

## Results

### Selection of Sources of Evidence

**Fig. 1:**
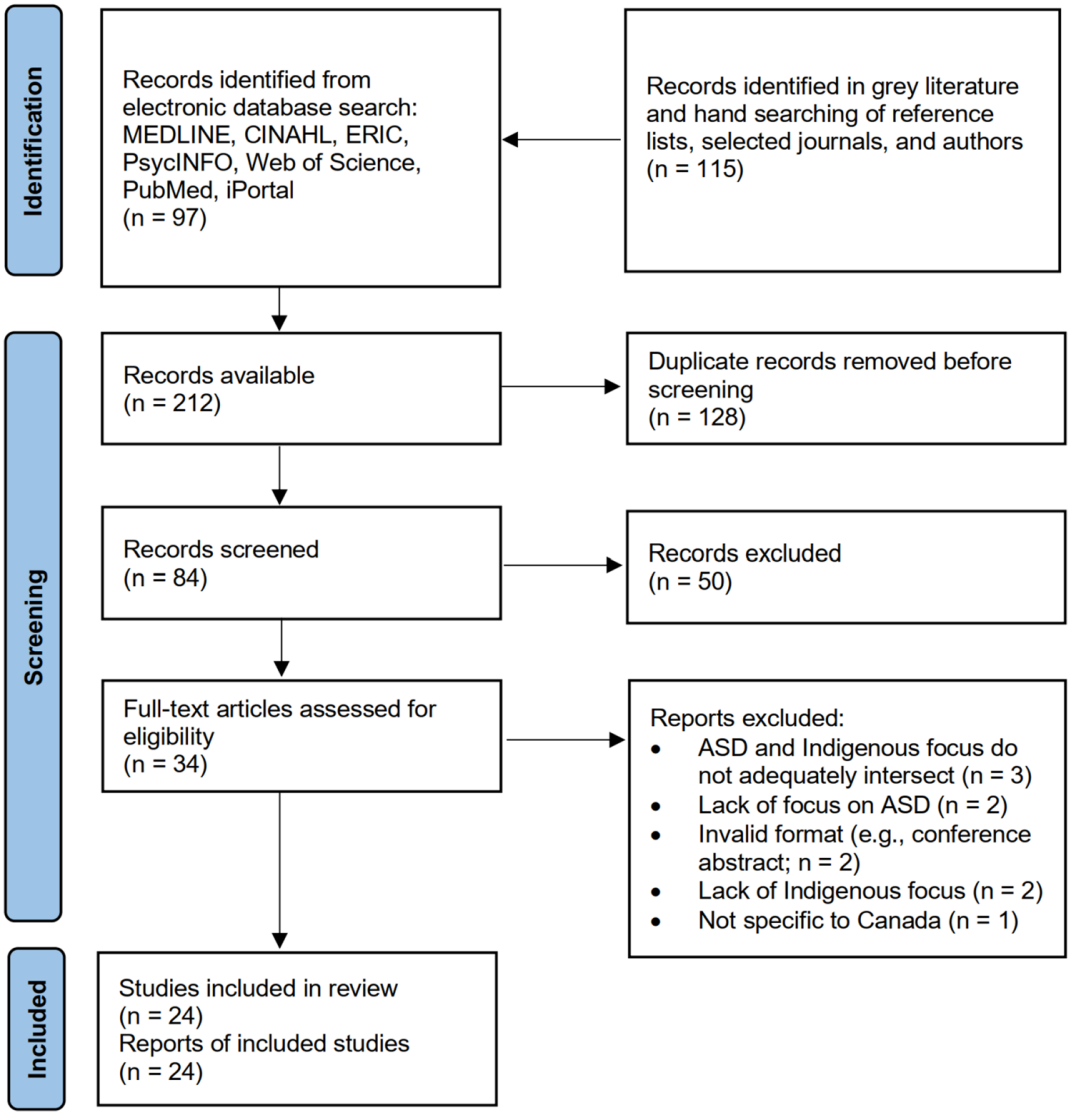
PRISMA flow diagram showing document retrieval and selection process

The initial database search yielded 212 articles. After removing 128 duplicates, 84 articles remained. Both reviewers (GB and TC) independently reviewed titles and abstracts of all 84 articles and screened out 50 articles. From the remaining sources (n = 34), the authors excluded 10 articles through full-text review as they did not meet the inclusion criteria (e.g., irrelevant study, lack of Canadian context). Thus, a total sample of 24 articles met the inclusion criteria for this review (see Fig. 1). Substantial interrater agreement (κ = 0.633) and high proportional agreement (81.81%) were achieved in the initial title and abstract screening. This level of concordance was sustained during the full-text review (κ = 0.655; 84.38% proportional agreement). Aggregate summaries of study characteristics (see Table 2) and quality (see Table 3) have been generated in tabular format.

**Table 2:** Characteristics of selected articles

Characteristic	Studies
	*n* (%)
Year of publication	
2006-2010	4 (16.67)
2011-2015	4 (16.67)
2016-2020	10 (41.67)
2021-2022	6 (25.00)
Peer-reviewed	
Yes	15 (62.50)
No	9 (37.50)
Type of publication	
Academic journal article	14 (58.33)
Peer-reviewed research methods case report	1 (4.17)
Grey literature: dissertation	1 (4.17)
Grey literature: policy, planning, or commentary	2 (8.34)
Grey literature: program evaluation or engagement summary	5 (20.83)
Grey literature: research poster	1 (4.17)
Research methodology type	
Peer-reviewed	
Qualitative research	4 (16.67)
Quantitative research	2 (8.33)
Literature reviews	6 (25.00)
Unapplicable (commentary & theory article)	3 (12.50)
Grey literature	
Qualitative project	3 (12.50)
Quantitative project	1 (4.17)
Mixed methods project	3 (12.50)
Unapplicable (literature search & program/resource guide)	2 (8.33)
Location of interest in article	
Canada	22 (91.67)
International (including Canada)	2 (8.33)
Indigenous focus (FNMI)	
All (first nations, metis, inuit)	14 (58.33)
First nations	9 (37.50)
First nations & metis	1 (4.17)
Self-identified indigenous authorship	
Yes	7 (29.17)
No	17 (70.83)
Indigenous involvement in developing & implementing study	
Full involvement	3 (12.50)
Substantial involvement	7 (29.17)
No involvement	10 (41.67)
Uncertain	4 (16.67)
Research situated within context of colonialism	
Yes	13 (54.17)
No	11 (45.83)
Discussion included strengths-based insights	
Yes	12 (50.00)
No	10 (41.67)
Unsure	2 (8.33)

**Table 3:** Indigenous quality assessment tool results from selected articles

Questions	Response type			
	Yes (%)	Partially (%)	No (%)	Unclear (%)
1. Did the research respond to a need or priority determined by the community?	7 (29.17)	10 (41.67)	3 (12.5)	4 (16.67)
2. Was community consultation and engagement appropriately inclusive?	5 (20.83)	3 (12.5)	13 (54.17)	3 (12.5)
3. Did the research have Indigenous research leadership?	4 (16.67)	5 (20.83)	12 (50.00)	3 (12.5)
4. Did the research have Indigenous governance?	3 (12.5)	0 (0.00)	20 (83.33)	1 (4.17)
5. Were local community protocols respected and followed?	4 (16.67)	1 (4.17)	14 (58.33)	5 (20.83)
6. Did the researchers negotiate agreements with regards to rights of access to Indigenous peoples existing intellectual and cultural property?	0 (0.00)	1 (4.17)	16 (66.67)	7 (29.17)
7. Did the researchers negotiate agreements to protect Indigenous ownership of intellectual and cultural property created through the research?	1 (4.17)	1 (4.17)	17 (70.83)	5 (20.83)
8. Did Indigenous peoples and communities have control over the collection and management of research materials?	1 (4.17)	3 (12.5)	18 (75.00)	2 (8.33)
9. Was the research guided by an Indigenous research paradigm?	4 (16.67)	1 (4.17)	18 (75.00)	1 (4.17)
10. Does the research take a strengths-based approach, acknowledging and moving beyond practices that have harmed Indigenous peoples in the past?	7 (29.17)	5 (20.83)	11 (45.83)	1 (4.17)
11. Did the researchers plan and translate the findings into sustainable changes in policy and/or practice?	6 (25.00)	9 (37.50)	8 (33.33)	1 (4.17)
12. Did the research benefit the participants and Indigenous communities?	3 (12.5)	4 (16.67)	5 (20.83)	12 (50.00)
13. Did the research demonstrate capacity strengthening for Indigenous individuals?	2 (8.33)	7 (29.17)	15 (62.50)	0 (0.00)
14. Did everyone involved in the research have opportunities to learn from each other?	8 (33.33)	3 (12.5)	9 (37.50)	4 (16.67)

### General Characteristics

Overall, most articles focused solely on Indigenous peoples and autism specifically in Canada, and only 2 studies (Shochet et al., 2020; Simpson, 2021) included an international focus (see Table 4). Within Canada, the majority had a pan-Indigenous focus (Antony et al., 2022; Burstyn et al., 2010; Canadian Academy of Health Sciences, 2022; Canadian Autism Partnership Project, 2017; Di Pietro and Illes, 2014; Di Pietro and Illes, 2016; El-Hayek, 2007; Gerlach et al., 2022; Inman, 2019; Shochet et al., 2020; Simpson, 2021; Stavropoulou-Kampoukou, 2019; Virginia Lane, 2022), with some focusing strictly on First Nations (Auerbach, 2007; Cowessess First Nation, 2021; Lindblom, 2014, 2016, 2017a, b; Manitoba First Nations Child and Family Services, 2017; Woodgate et al., 2013), and one was Métis in focus (which also included First Nations) (Thompson, 2012). None of the articles were explicit on focusing on Inuit populations. Of the 24 articles, seven had self-identified Indigenous authorship or co-authorship, and none had self-identified Autistic authors.

**Table 4:** Details of articles included in scoping review

Author (s) and publication date	Self-Identified Indigenous Authorship	Sample/Participants	Aim (s)
*Previous reviews related to ASD and Indigenous communities in Canada*			
^†^Lindblom (2014)	No	17 sources (including both academic and grey literature) across the United States, the Netherlands, Ghana, Korea, Australia, and Canada, published 2003 to 2013.	To examine possible explanations in literature for the under-detection of ASD among Indigenous communities.
^†^Di Pietro and Illes (2014)	No	52 sources published in Canada between 1981 to 2011.	To map the landscape of research on ASD, cerebral palsy, and FASD in Canadian Aboriginal children.
^†^Shochet et al. (2020)	No	7 academic articles and 11 psychosocial resources/programs across the United States, Canada, Australia, and New Zealand.	To identify psychosocial programs and resources developed world-wide for Indigenous people with ASD and their caregivers.
Stavropoulou-Kampoukou (2019)	No	Final number of total documented included unspecified, but consisted of journal articles and grey literature published from 2009 to 2019.	To investigate literature on Indigenous populations with developmental disabilities, as well as delivery and access to services.
^†^Simpson (2021)	No	24 journal articles across Canada, the United States, Australia, and New Zealand, published from 2012 to 2020.	To compare literature internationally regarding culturally responsive interventions aimed at: (1) promoting strong cultural identity, and (2) safeguarding Indigenous and Autistic people from stigmatization, misrepresentation, and identity erasure.
^†^Antony et al. (2022)	Yes	9 sources across Canada and the United States, published between 2004 and 2021.	To provide a framework for understanding the conceptualization of ASD in Indigenous Peoples and the interactions between racialized Autistic peoples and the Criminal Justice System.
^†^Gerlach et al. (2022)	Yes	19 sources across Canada published between 2007 and 2020.	To critically analyze key themes on ASD and the provision of ASD-related services with Indigenous children and families in Canada.
*Community and policy reports*			
Auerbach (2007)	Yes	First Nations schools in BC receiving provincial special education program funding (N = 106) with over 5,608 total students.	To determine the prevalence of special needs students with learning/social needs in First Nations schools, as well as summarize inclusion of special needs students in an educational district’s existing programs/services.
Woodgate (2013)	No	103 families, service providers, policymakers, elders, and other key informants across Manitoba.	To examine: (1) how Indigenous families assign meaning to childhood disability, (2) their health & social service use in context, (3) how Indigenous families position childhood disability at different levels to influence participation in daily life, (4) how ecological understandings and considerations contribute to participation in life.
Manitoba First Nations Child and Family Services (2017)	Yes	Engagement sessions across Manitoba with 20 First Nations communities, 4 public townhalls, 1 youth sessions, two provincial child welfare agencies, provincial and federal governments, Chief’s Task Force, Elders Gathering, and the Grandmothers Council.	To pursue First Nations-led local child welfare system reform through a community engagement process with local Indigenous leadership and communities.
Canadian Autism Partnership Project (2017)	No	4,963 Canadians across both urban and rural settings, including government officials and public participants.	To make a case to the government for a national autism partnership, summarizing extensive research efforts and engagement with Canadians on systemic needs relating to ASD.
Canadian Academy of Health Sciences (2022)	Yes	Pan-Canadian online respondents, interview participants consisting of Indigenous Autistic individuals, their family members, and service providers.	To provide a preliminary overview on perspective of Indigenous peoples related to ASD and highlight the inequities experienced.
Cowessess First Nation & Autism Resource Centre (2021)	Yes	Elders/Knowledge Keepers and families from one First Nations community in southern Saskatchewan, participating in a partnership with an Autism resource centre.	To explore and begin to address gaps in culturally appropriate autism resources for Indigenous people, families, and communities.
Canadian Academy of Health Sciences (2022)	Yes	–	To directly inform the development of a national autism strategy in Canada, as well as future public policy in this area.
*Primary research and commentaries*			
^†^Ouellette-Kuntz et al. (2006)	No	Children under 15 years of age who resided in Manitoba or PEI during 2002 with a diagnosis of PDD or Autism, identified by a provincial government support program.	To estimate the prevalence of pervasive developmental disorders within two Canadian provinces and compare/contrast characteristics of diagnosed cases between the two regions.
^†^El-Hayek (2007)	No	–	To examine implications of mercury environmental contamination in Arctic Canada and its impacts on Indigenous communities residing in the region.
^†^Burstyn et al. (2010)	No	All singleton live births (N = 273, 343) in Alberta between 1998 and 2004, and those receiving follow-up services up until 2008 identified through physician billing records.	To estimate incidence and prevalence of ASD among a population-based birth cohort in Alberta and to explore whether maternal traits and obstetric complications are associated with ASD in this population.
^†^Thompson (2012)	No	Teacher candidates (n = 16) enrolled in the final semester of a post-secondary program in Regina, Saskatchewan, who participated in a novel social justice-oriented curriculum.	To develop a social justice-oriented and inclusive pedagogy that situates traditional individualised views of disability in light of three alternative understandings: an education perspective of disability studies, an Indigenous view of disability, and a perspective based on the autism pride/autism-as-culture movement.
^†^Di Pietro and Illes (2016)	No	Researchers (n = 8) who have 15 years’ experience working in and with Indigenous communities across Alberta, BC, Manitoba, including 5 pediatricians, 2 Indigenous health researchers and policymakers, and 1 social worker.	To investigate among health researcher working with Indigenous communities: (1) the lack of research within the Indigenous context on ASD and cerebral palsy, (2) ethical and social ramifications of this disparity, (3) recommendations for change.
^†^Lindblom (2016)	No	–	An educational case that gives a personal account of doing ethnographic research with Indigenous peoples.
^†^Lindblom (2017a)	No	5 Indigenous children with ASD, family members, and school staff, in both rural and urban BC.	To examine the use and meaning of music for five First Nations children diagnosed with autism spectrum disorder in British Columbia, Canada.
^†^Lindblom (2017b)	No	2 Indigenous children with ASD and their family and school staff in BC, with differing residences (1 on reserve and 1 off reserve).	To investigate the use of music interventions and activities to overcome obstacles or facilitate social inclusion, using two cases of young First Nations people diagnosed with autism, in British Columbia, Canada.
Lindblom (2017c)	No	5 Indigenous children with ASD, family members, and school staff, in both rural and urban BC.	To uncover how an ASD diagnosis is perceived, how music is used, and the role of music to facilitate inclusion for First Nations children with autism in BC, Canada.
^†^Inman (2019)	No	–	To identify, analyze, and contrast the discourses for ASD and FASD across Canada pertaining to Indigenous communities.

^†^Published in a peer-reviewed journal

*BC* British Columbia; *PEI* Prince Edward Island; *PDD* Pervasive Developmental Disorders; *FASD* Fetal Alcohol Spectrum Disorders

Of the 24 articles, 15 were peer reviewed (Antony et al., 2022; Burstyn et al., 2010; Di Pietro & Illes, 2014; Pietro & Illes, 2016; El-Hayek, 2007; Gerlach et al., 2022; Inman, 2019; Lindblom, 2014, 2016, 2017a, b; Ouellette-Kuntz et al., 2006; Shochet et al., 2020; Simpson, 2021; Thompson, 2012) and 9 were considered grey literature (Auerbach, 2007; Canadian Academy of Health Sciences, 2022; Canadian Autism Partnership Project, 2017; Cowessess First Nation, 2021; Manitoba First Nations Child and Family Services, 2017; Stavropoulou-Kampoukou, 2019; Virginia Lane, 2022; Woodgate et al., 2013; Lindblom, 2017c). In the following section, we systematically address each research question based on the reviewed literature.

### Research Question 1: What is Known About Autism in Indigenous Communities in Canada Based on the Literature?

Overall, there was a limited literature on autism and Indigenous peoples in Canada. Several articles pointed toward the need for data and research to be done on autism in Indigenous communities, but there was a lack of clarity about who should conduct this research and how the research was to be conducted.

There were a total of five reports, two of which were community reports (Cowessess First Nation, 2021; Manitoba First Nations Child and Family Services, 2017), and 3 that have a national focus (Canadian Academy of Health Sciences, 2022; Canadian Autism Partnership Project, 2017; Virginia Lane, 2022). Only three articles were considered to have full involvement of Indigenous peoples in the planning and execution of the research (Cowessess First Nation, 2021; Gerlach et al., 2022; Manitoba First Nations Child and Family Services, 2017). Of these three articles, two were community reports (Cowessess First Nation, 2021; Manitoba First Nations Child and Family Services, 2017).

Most of the articles had a pan-Indigenous focus within Canada, with an exclusive focus on First Nations being second most common. When using a pan-Indigenous approach, it is important to use caution as not all findings can be applied broadly across various Indigenous communities or populations. Indigenous peoples across Canada have unique historical and cultural differences that may be influenced by language, legislation, and geography. For example, in Inman’s (2019) article on both autism spectrum disorder (ASD) and fetal alcohol spectrum disorder (FASD), there are sweeping statements made that generalize Indigenous populations. While it is understood that FASD does affect Indigenous populations at a higher level, Indigenous people are not a monolith and while ASD may be present, ASD could be misdiagnosed as FASD, at varying rates among different First Nations, Métis, or Inuit populations due to these sweeping statements.

The two community reports provided valuable insights regarding the experiences of autism in their respective communities. For example, the Cowessess First Nation community report (2021) outlined Nehiyaw (Plains Cree) cultural understanding of autism. Although the community report was not peer reviewed, it provides important insights into an Indigenous, specifically Nehiyaw, worldview on autism. The report explored several culturally informed definitions of autism outlined below,

- ka-kamawaci-iyinisit (given a unique quiet spiritual intelligence).
- pihtos-mânitonihk-iyinisit (given a different way of thinking in one’s own spiritual intelligence).
- ê-mihkosit pihtos mânitonicihkan (given a different way of spiritual thinking).
- ê-mihkosit pihtos ê-si-waskawiht (given the gift of moving a different way or being [characteristic/behavior]) (p. 3).

### Research Question 2: What are the Areas of Focus of Published Research on Autism and Indigenous Peoples in Canada?

All but two articles had some focus on program delivery relative to autism. The programs described included special education (Auerbach, 2007; Manitoba First Nations Child and Family Services, 2017), barriers to diagnoses, early diagnosis, and misdiagnosis (Burstyn et al., 2010; Canadian Academy of Health Sciences, 2022; Canadian Autism Partnership Project, 2017; Inman, 2019; Lindblom, 2014; Ouellette-Kuntz et al., 2006), lack of culturally informed services and cultural understandings of autism (Antony et al., 2022; Lindblom, 2017a; Shochet et al., 2020; Thompson, 2012), negative systemic impacts and stereotyping (Gerlach et al., 2022), and inequitable access to services and funding (Lindblom, 2017b; Simpson, 2021).

A key finding explored in the articles was discrepancies related to equitable access of Indigenous children and their families to services such as diagnostic assessment and interventional support, and the influence of geography and legal status. Specifically, Lindblom (2017c) argues that First Nations children who live on reserve have less access to resources and support than those who do not live on reserves. There are several factors for the lack of services on reserves, but Lindblom (2017a, b, c) argues the most substantial barrier entails jurisdictional disputes between the provincial and federal government.

The literature generally offered some focus on the experiences of autism, with the exception of five articles (Burstyn et al., 2010; Di Pietro & Illes, 2014; El-Hayek, 2007; Ouellette-Kuntz et al., 2006; Stavropoulou-Kampoukou, 2019). Specifically, Di Pietro’s & Illes (2016) article demonstrated the realities of being a member of the Indigenous Autistic community, but also outlined a way to move forward, stating: “all participants affirmed that engagement in autism spectrum disorder health research can only take place if the outcomes of research directly benefit the communities. Such outcomes must be focused on improving access to health care and services. Under these circumstances, they predicted that trust in non-Aboriginal neurodevelopmental disorder researchers would follow” (p. 246). Lindblom’s (2017b) explored the challenges and negative experiences of raising an autistic child on a reserve and how some families traveled upwards of 300 km to get a diagnosis. However, the author also highlighted how incorporating First Nations culture, such as drumming, into the child’s life promoted better overall wellbeing and provided a space for the family to feel connected.

### Research Question 3: What is the Quality of Research on Autism in Indigenous Communities in Canada?

A dearth of research using primary data was observed in this review, with just under half of all included articles (n = 11) being a review, report, or commentary. Of the remaining articles which reported primary research, six were peer-reviewed and were not led by Indigenous people. Primary research identified in this review, such as community reports, tended to use relationship-based methodologies and frameworks more culturally accommodating to Indigenous communities. Examples included ethnography (Lindblom, 2014; Lindblom, 2017a, b, c) and *Touchstones of Hope* (Manitoba First Nations Child and Family Services, 2017) which comprises a set of principles guiding reconciliation, fostering relationships, and facilitating conversations on systems reform.

None of the articles met all the criteria for the IQAT, with most of the articles scoring low quality. The highest scoring article was positively scored in 11 of the 14 IQAT categories/questions (Manitoba First Nations Child and Family Services, 2017), followed by 10 out of 14 (Cowessess First Nation, 2021), and then 7 out of 14 (Lindblom, 2017a; Woodgate et al., 2013). Overall, two studies were assessed as high quality, three as moderate quality, and 19 as low quality. Two criteria that were met more consistently were ‘responding to the need or priority by the community’ (yes = 7, partially = 10), and whether the researchers planned on translating their findings to sustainable changes in policy and practice (yes = 6, partially = 9).

In the IQAT, criteria 6 (yes = 0, partially = 1), 7 (yes = 0, partially = 1) and 8 (yes = 1, partially = 3) all focus on Indigenous research and data ownership. Articles consistently scored poorly on these criteria. Articles generally scored the lowest on IQAT questions on the inclusion of governance (no = 20) and use of an Indigenous research paradigm (no = 18). The criteria on whether the research directly benefitted Indigenous communities was the most challenging to score in the appraisal (unclear = 12).

## Discussion

Our aim was to explore, gather, collate, and analyze all the known literature on autism in Indigenous communities specific to Canada. Overall, we found that not only is there a severe gap in the literature on autism and Indigenous communities, but also the quality of the literature is low from an Indigenous standpoint, based on analysis using the IQAT. When engaging with the literature, it is important to maintain a critical lens and recognize that the findings may not be truly representative of what is happening in Indigenous communities, for example, while there is some discussion of the obstacles to getting a diagnosis the lived experience of being an Indigenous Autistic has not been explored accurately enough to find equitable solutions to the obstacles. This review also shows there is critical need for primary research that is community-led across various areas and sectors of autism scholarship and practice. Indigenous Autistics and communities are largely being left out of the research process; hence, the results of current research can be problematic. Funding organizations and researchers need to recalibrate their research approaches to be more inclusive. Until that happens, it is difficult to have confidence that the results are reflective autistic Indigenous people’s wants and needs. It is not surprising that the Indigenous community research or Indigenous led studies scored better on the IQAT. This can be attributed to the communities being able to lead the research and seek results and through this process, reflect on the true wants and needs of the community.

Our findings are like those of another scoping review completed recently in the context of Indigenous autism and Canada. Although Gerlach et al’s (2022) scoping review examined similar literature in a similar context, yet the results of that review and the current review have some key differences. Gerlach’s review explored Indigenous autism from a social work perspective with the following research questions; “what is known about autism diagnosis and prevalence in Indigenous communities in Canada?; What is known about how autism is perceived by Indigenous peoples in Canada?; What is known about the intersections of autism, the child protection system in Canada and Indigenous communities?” (p. 7). While these research questions are resonant and complementary to our review (and yielded may of the same studies/articles), different results emerged. Whereas the current review was Indigenous led we also assessed the quality of the research, Gerlach’s review explored content with little examination of the quality. This is an important distinction because when policy planners or program personnel are seeking to make decisions related to autistic Indigenous individuals and their families, this current review (via the application of IQAT and its results) offers caution in applying the current Indigenous autism literature in Canada due to quality limits in an Indigenous context. The gaps identified in this review strongly call for more Indigenous led and high-quality research in autism.

It is not surprising that each of the IQAT criteria were aligned with research agreements, ownership, and control of the research. This finding contrasts with western research which often imposes power imbalances that benefit the researcher and not the researched. The relatively poor scores on the IQAT generally indicate an overall critical need for Indigenous-led research on autism in Canada. As Indigenous autism research continues to emerge, academic institutions and other scholarly bodies (e.g., funders) need to provide equitable opportunities to Indigenous peoples. Within this shift, Indigenous Autistics can increasingly lead this research or work together as full partners. The lack of Indigenous input can be attributed to many factors including the lack of awareness about autism in First Nations communities, lack of research capacity for Indigenous people, and/or ongoing mistrust of research institutions (Blanchet Garneau et al., 2021) While Gerlach’s et al. review provides important insights, ensuring the literature is reliable, credible, and applicable is critical. There have been some improvements made in the past decade to working with Indigenous people in a research context and this can be attributed to Truth and Reconciliation Commission’s (TRC) Calls to Action (*Truth and Reconciliation Commission of Canada : Calls to Action.*, 2015) and the Tri-Council Policy Statement (TCPS) for Ethical Conduct for Research Involving Humans and includes the chapter Research Involving the First Nations, Inuit and Métis Peoples of Canada (Tri-Agencies, 2016). As Canada moves forward in era of reconciliation researchers and academic institutions must prioritize the perspectives of Indigenous people throughout the entire research process.

There have been similar reviews done in other countries with the two most notable being in Australia and New Zealand. In Bailey & Arciuli’s (2020) scoping review, the authors explored autism in Indigenous communities in Australia. They identified similar barriers to what is found in Canada, including less access to diagnostic services, misdiagnoses, and lack of data on autism such as prevalence, with recommendations to support improved quality of life for Indigenous Autistics. Moreover, in a scoping review examining autism in Māori communities New Zealand, Tupou et al. (2021) determined cultural understandings similar to what the Cowessess First Nation community report explored. Tupou and colleagues (2021) further observed the need for improved diagnostic services and supports. Notably, these reports exemplify what can be achieved if the community is able to take the lead in their own research interests. They importantly seemed to represent the community in a way that traditional peer reviewed articles seemingly do not. When contrasting the findings of this scoping review with the others done in Canada, Australia, and New Zealand, the emergent themes are similar across reviews, including challenges to diagnosis, different understandings of autism, and the lack of research in this area. All these scoping reviews highlighted the critical need for culturally informed services and supports for Indigenous Autistics.

Finally, Canada has a history of human rights abuses against Indigenous children including children with disabilities. In 2007 the First Nations Child and Family Caring Society (FNCSFCS) of Canada and the Assembly of First Nations (AFN) brought forward allegations that the federal government had discriminated against First Nations children under the *Canadian Human Rights Act* (Blackstock, 2016). The FNCFCS and AFN successfully argued the crown discriminated against First Nations children in a flawed child welfare system, which includes disability services and supports. In 2016, the Human Rights Tribunal agreed that Canada was indeed discriminating based on race, yet until recently the federal government of ignored and failed to comply with this ruling. As Canada attempts to move forward in reconciliation with Indigenous peoples, it is important to recognize the historical and contemporary injustices Indigenous children continue to face.

Despite the challenges noted above, Indigenous communities and families in Canada have shown strength and resilience. Families continue to show up and support Autistic family members. Indigenous communities are the product of centuries of ongoing destruction that has its roots in colonialism, yet they are also the product of resiliency that has been passed down through the generations. Indigenous people across Canada have endured systemic and day to day problems, yet they are still able to practice their culture, speak their languages, and stay connected through kinship. These strengths are means by which Indigenous families can support Autistic relatives and those in their communities, and through strength-based lens this review aimed to emphasize the importance of Indigenous led work to eventually better support Indigenous families (Bryant et al., 2021).

### Limitations

Scoping reviews encapsulate a broad scope and volume of literature. While we had an extensive and thorough search strategy, we recognize that much of what was yielded in this review reflected grey literature. The adapted Indigenous IQAT was specifically developed to apply to studies about Indigenous peoples. However, we recognize that it was developed in Australia, hence has cultural influences that differentiate from a local context. However, despite these potential limitations in the IQAT, its implementation exceeds what is typical of a scoping review and appeared to add important study evaluation. Moreover, we adapted that tool to our context. In applying the IQAT for determining individual study quality, we acknowledge that this appraisal only accounts for what is present in a manuscript and thus relevant details that are not published may not be accounted for. Although most articles were unclear in some areas of quality adjudication, we acknowledge that there could have been information that was left out within the writing, review, and publication processes. Finally, due to the limited body of literature, this review used a pan Indigenous approach, and we recognize this as a limitation of the review. Future work focused on the specificity of individual communities would be beneficial.

### Recommendations

A key finding from this review was the lack of Indigenous-led peer reviewed research on autism in Indigenous communities. While this finding is concerning, it also presents as a unique and important opportunity for advancing community research capacity. Indigenous communities have often been on the receiving end of research. Creating space for Indigenous people and communities to lead their own research in the underserved area is of priority. Building meaningful partnerships with Indigenous communities and providing opportunities for community members to lead and guide the research in meaningful ways will provide much needed literature that truly reflects the wants and needs of Indigenous communities.

The community report done by Cowessess First Nation provides powerful insights into cultural understandings of autism. Moving forward, it is important to ensure opportunities to explore different understandings of autism that may challenge the predominant western approaches (e.g., medical model). Autism is often framed as a deficit and something to be solved, but the Cowessess report demonstrated that an Indigenous approach can be more inclusive and less “othering”. The Indigenous approach can be used to address the stigma that is often associated with an autism diagnosis and potentially a challenge to a family in seeking a diagnosis.

Antony et al’s (2022) recent article outlines the following five actionable items for improving the lives of autistic Indigenous people: (1) Develop an Indigenous autism engagement framework that recognizes and honors the diversity of Indigenous peoples across Canada; (2) Assess the barriers to healthcare including diagnostic assessments, services provision, socio economic considerations, and overall mistrust of the health care system; (3) Partner with Indigenous communities and organizations to collect data and evidence to understand autism prevalence and the lived experience of autism in Indigenous communities; (4) Explore what Indigenous led and culturally safe services, assessments, and interventions would look like; (5) Provide equitable funding across Canada for Indigenous communities and organizations to provide culturally safe autism services, appropriate autism assessments, autism awareness and education (p. 08). If implemented, these recommendations would move us forward in our quest for equitable opportunities for Indigenous autistic people and their families and potentially ameliorate some of the challenges Indigenous communities and families currently face.

## Conclusions

To our knowledge, this is the first scoping review on autism and Indigenous people in Canada that is fully led by Indigenous people and has been brought back to the community for them to give their perspective. We also uniquely used the Indigenous Quality Assessment Tool (IQAT) to measure the literature from an Indigenous perspective. Future research in the area Indigenous peoples and autism in Canada must have substantial Indigenous input that respects the unique worldviews, rights, and knowledges. This is critically important as we now understand differences in how autism is perceived through an Indigenous lens as opposed to a western lens. Finally, this review shows that there is an urgent and critical need for Indigenous-led and high-quality research that explores both prevalence and lived experiences of autism in Indigenous communities across Canada.
